# Demographics of Pediatric OHCA Survivors With Postdischarge Diseases: A National Population-Based Follow-Up Study

**DOI:** 10.3389/fped.2019.00537

**Published:** 2020-01-24

**Authors:** Tsung-Han Lee, I-Cheng Juan, Hsiu-Ying Hsu, Wen-Liang Chen, Cheng-Chieh Huang, Mei-Chueh Yang, Wei-Yuan Lei, Chih-Ming Lin, Chu-Chung Chou, Chin-Fu Chang, Yan-Ren Lin

**Affiliations:** ^1^Department of Emergency Medicine, Changhua Christian Hospital, Changhua City, Taiwan; ^2^Department of Biological Science and Technology, National Chiao Tung University, Hsinchu, Taiwan; ^3^Department of Nursing, Dayeh University, Changhua City, Taiwan; ^4^Department of Nursing, Changhua Christian Hospital, Changhua City, Taiwan; ^5^Department of Neurology, Changhua Christian Hospital, Changhua City, Taiwan; ^6^Department of Social Work and Child Welfare, Providence University, Taichung City, Taiwan; ^7^Department of Medicinal Botanicals and Health Applications, Dayeh University, Changhua City, Taiwan; ^8^School of Medicine, Kaohsiung Medical University, Kaohsiung City, Taiwan; ^9^School of Medicine, Chung Shan Medical University, Taichung City, Taiwan

**Keywords:** children, OHCA, survival, postdischarge, functional, neurological

## Abstract

**Background:** Postdischarge diseases (PDDs) have been reported for adult survivors of out-of-hospital cardiac arrest (OHCA). However, the detailed demographics of pediatric OHCA survivors with PDDs are not well-documented, and information regarding functional survivors is particularly limited. We aimed to report detailed information on the PDDs of survivors of traumatic and non-traumatic pediatric OHCA using a national healthcare database.

**Methods:** We retrospectively obtained data from the Taiwan government healthcare database (2011–2015). Information on the demographics of traumatic and non-traumatic pediatric OHCA survivors (<20 years) was obtained and reported. The patients who survived to discharge (survivors) and those classified as functional survivors were followed up for 1 year for the analysis of newly diagnosed PDDs. The time from discharge to PDD diagnosis was also reported.

**Results:** A total of 2,178 non-traumatic and 288 traumatic OHCA pediatric cases were included. Among the non-traumatic OHCA survivors (*n* = 374, survival rate = 17.2%), respiratory tract (*n* = 270, 72.2%), gastrointestinal (*n* = 187, 50.0%), and neurological diseases (*n* = 167, 49.1%) were the three most common PDD categories, and in these three categories, the majority of PDDs were atypical/influenza pneumonia, non-infective acute gastroenteritis, and generalized/status epilepsy, respectively. Among the traumatic OHCA survivors (*n* = 21, survival rate = 7.3%), respiratory tract diseases (*n* = 17, 81.0%) were the most common, followed by skin or soft tissue (*n* = 14, 66.7%) diseases. Most functional survivors still suffered from neurological and respiratory tract diseases. Most PDDs, except for skin or soft tissue diseases, were newly diagnosed within the first 3 months after discharge.

**Conclusions:** Respiratory tract (pneumonia), neurological (epilepsy), and skin or soft tissue (dermatitis) diseases were very common among both non-traumatic and traumatic OHCA survivors. More importantly, most PDDs, except for skin or soft tissue diseases, were newly diagnosed within the first 3 months after discharge.

## Introduction

Out-of-hospital cardiac arrest (OHCA) in children is rare, and its outcome is very poor. Several studies have reported an OHCA survival rate of 6.7–10.2% and that at most 4% of patients have intact or functional neurological outcomes (1–7). Most pediatric survivors will have previously been subjected to high levels of physical or psychological stress from the resuscitation process and treatment procedures or even from the effects of high-dose drugs during their hospital stay (8–11). Furthermore, postcardiac arrest syndrome and individual essential diseases (related to cardiac arrest) may potentially not only increase mortality during admission but also induce short/long-term postdischarge diseases (PDDs) (i.e., neurological defects, hypoxia-related organ dysfunction, and opportunistic infections) (10, 12–16).

In adult OHCA patients, several health economic studies have noted that the cost of long-term care for non-functional survivors is quite high. For example, the cost of long-term care for each survivor would be approximately USD$102,000 and USD$75,000 (per year) on average in the UK and USA, respectively (17, 18). More importantly, the cost would obviously increase once a PDD occurred, especially if the disease was not noticed in a timely manner or not well-controlled (15, 19, 20). Therefore, a detailed understanding of the demographic features of PDDs that may occur in OHCA survivors will help primary physicians perform appropriate clinical assessments. Some demographic studies focusing on post-resuscitation evaluations have reported that infections, heart failure, and gastrointestinal hemorrhage were the most common PDDs in adult OHCA survivors (21, 22). However, because body proportion, maturity, and recovery capability are likely to differ substantially between adult and pediatric patients, the conditions in which PDDs occur might also be different (23, 24). Unfortunately, studies focusing on PDDs in pediatric survivors are still limited.

A few epidemiological studies have emphasized the importance of post-resuscitation care and have reported the long-term survival rates and neurological outcomes of pediatric OHCA patients (25–28); however, detailed information regarding PDDs is not available, especially regarding the disease onset times, which are unclear. In this study, we aimed to report detailed information on the PDDs of traumatic and non-traumatic pediatric OHCA survivors with a 1-year follow-up time using a national healthcare database.

## Methods

### Database

This is a national database study. Data were obtained from the Taiwan government healthcare database (National Health Insurance program). This database covered almost 100% of the population (22 million people) in Taiwan during the study period (January 2011 to December 2015). With the government's permission, data from the database (de-identified data) can be retrospectively extracted and analyzed for the purpose of scientific research.

#### Patient Privacy Policy of Database

To protect personal privacy, data are not allowed to be revealed if the number of patients is lower than three (potential risk of identification). The data output was supervised by the Taiwanese government.

### Ethics Statement

This study followed the Strengthening the Reporting of Observational Studies in Epidemiology guidelines. The cardiac arrest events and outcomes (definition of OHCA: survival to discharge) of the patients adhered to the Utstein guidelines (29). The protocol of this study was approved by the institutional review board (IRB) of Changhua Christian Hospital (central Taiwan). Since, this is a retrospective database study and since all information were secondary data (de-identified), informed consent was not needed under the IRB guidelines:

1. Full name of the ethics committee: institutional review board of Changhua Christian Hospital, Changhua, Taiwan.
2. Consent procedure: All information in this database were secondary data (de-identified); no formal consent was necessary (permission code 180802).
3. Any additional considerations of the study in cases where vulnerable populations were involved, for example, minors, persons with disabilities, or endangered animal species: there were no additional considerations for vulnerable populations under the IRB permission.

### Study Setting and Population

**Figure 1:**
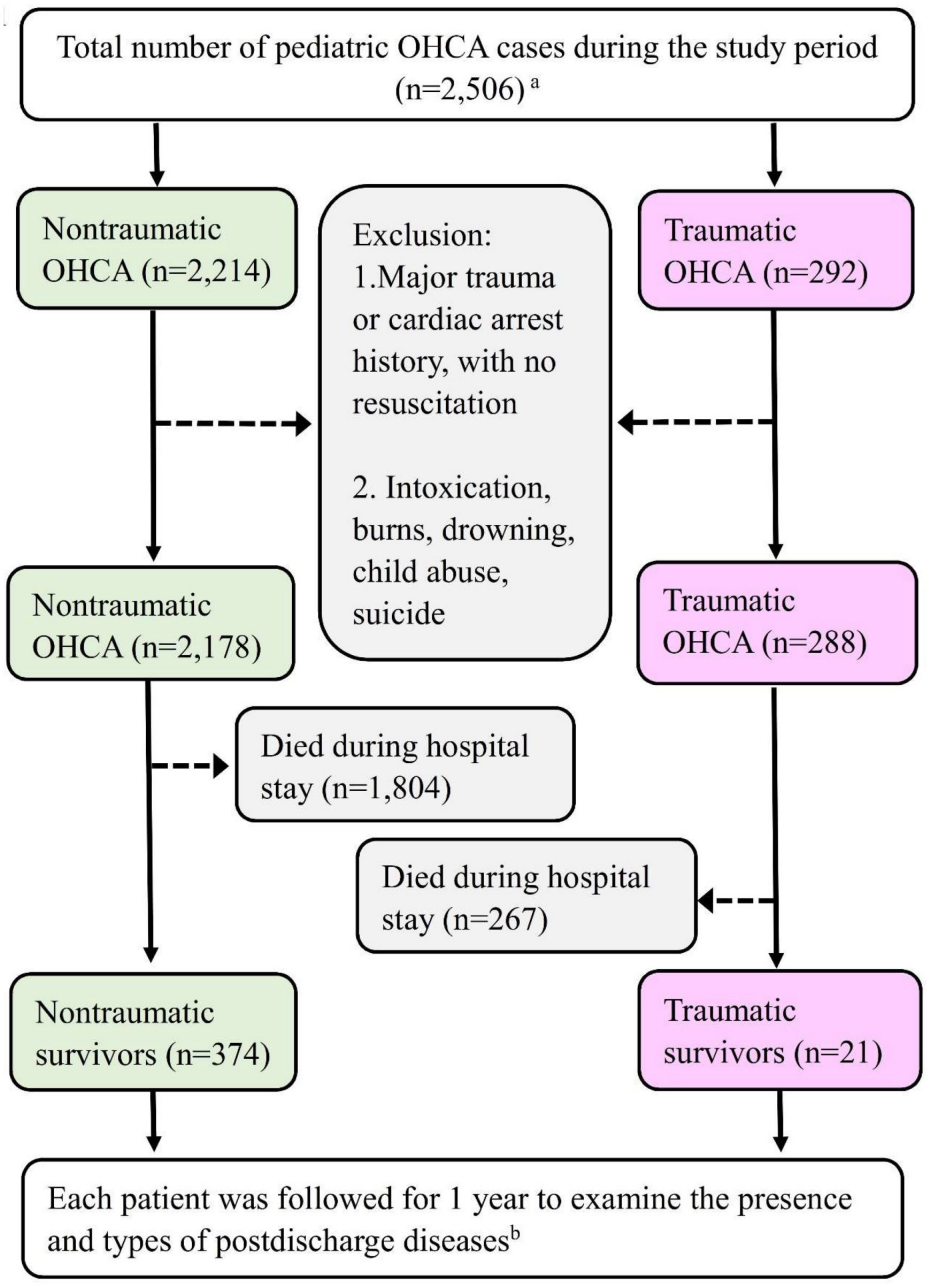
The selection principle and primary outcomes of the traumatic and non-traumatic OHCA survivors. Detailed information on the exclusion criteria is provided in the Methods section. ^*a*^*The pediatric population is defined as patients aged younger than 20 years*. ^*b*^*The postdischarge diseases are defined as diseases that are newly diagnosed after hospital discharge*.

All information regarding the OHCA pediatric cases were retrospectively obtained from the database from January 2011 to December 2015. Patients who suffered from OHCA but did not have any emergency department (ED) visits or treatment records were not included [i.e., declared dead by emergency medical services (EMS) personnel]. The selection flow chart is shown in Figure 1. Each traumatic or non-traumatic OHCA survivor (surviving to discharge) was followed up for 1 year to determine the frequencies of the newly diagnosed PDDs.

#### Inclusion Criteria

Patients who met all of the following characteristics during the study period were included:

1. Pediatric patients who had suffered their first non-traumatic or non-traumatic OHCA and been diagnosed by ED physicians, with OHCA defined using the International Classification of Diseases, 9th revision, clinical modification (ICD-9-CM) codes 798-799 and 427.5.
2. Pediatric patients who had received ED resuscitation.

#### Exclusion Criteria

Patients with the following conditions were excluded:

1. Patients with any history of cardiac arrest (traumatic/non-traumatic) or major trauma before the study began.
2. Patients who did not receive any EMS or ED resuscitation (including a do-not-resuscitate order).
3. Non-pediatric patients (aged > 20 years).
4. Patients with incomplete medical records.
5. Patients whose insurance was terminated during the study period.
6. Intoxication, burn, drowning, or criminal investigation cases (child abuse, suicide, or murder).

#### Definition of Traumatic and Non-traumatic OHCA

Traumatic OHCA patients were defined as having OHCA caused by trauma (major diagnosis including the ICD-9-CM codes 800-809, 810-819, 820-829, 830-839, 850-854, 860-862, 863-869, 900-904, 925-929, 940-949, and 950-957). The remaining patients were classified as non-traumatic OHCA.

#### Definition of Survivors and Functional Survivors

Patients who survived to hospital discharge (not including those who chose to leave before discharge) were defined as survivors. Survivors were classified as functional survivors if they met all of the following characteristics after discharge: (1) no long-term unconsciousness, (2) no need for long-term respirator support, (3) no need to be bed-ridden in the long term, and (4) no need to depend on others for long-term support (nasogastric tube feeding, indwelling bladder catheter, or tracheostomy). This study did not use the Pediatric Cerebral Performance Category Scale (PCPCS) as the principal criterion for evaluating survivors primarily because the PCPCS was not routinely or regularly used to evaluate each survivor after discharge.

#### Definition of Comparison Groups I and II

The cases in the comparison groups were obtained from the general population of the same national database (three comparison patients for each survivor). There were two comparison groups, and these groups were composed of patients that were age- and sex-matched to the patients in the non-traumatic (as group I) and traumatic (as group II) OHCA groups.

#### Definition and Classifications of PDDs

The PDDs in this study were defined as newly diagnosed diseases after hospital discharge in OHCA survivors. There were two major reasons that motivated us to analyze the PDDs. First, since the survivors had been subjected to higher levels of physical/psychological stress than the general population, we suspect that the OHCA survivors would be more likely to face various complications, especially after being discharged from the hospital (without intensive care). Second, multiple studies have reported PDDs in adult OHCA survivors (i.e., infections, heart failure, and gastrointestinal hemorrhage); however, the understanding of the PDDs of pediatric OHCA survivors is still poor. Information on PDDs was obtained from postdischarge medical records (including data from the follow-up outpatient department, data from ED visits, data from physician-diagnosed home care, and data from clinics). These diseases were divided into eight major categories: (1) respiratory tract diseases [pneumonia, acute upper respiratory infection (URI), acute bronchitis, etc.], (2) gastrointestinal diseases (acute gastroenteritis, ileus, irritable bowel syndrome, etc.), (3) neurological diseases (epilepsy, paralysis, cerebral palsy, etc.), (4) skin or soft tissue diseases (dermatitis, cellulitis, tinea corporis, etc.), (5) eye/ear diseases (conjunctivitis, hordeolum, otitis, etc.), (6) psychiatric diseases (attention deficit hyperactivity disorder, borderline personality disorder, etc.), (7) cardiovascular diseases (cardiac arrythmia, hypertension, etc.), and (8) urinary system diseases (urinary tract infection, cystitis, bladder dysfunction, etc.). Finally, the diseases of comparison groups (general pediatric population, matched with age and sex) were also reported.

### Study Protocol

The final population included 374 non-traumatic and 21 traumatic OHCA survivors. Comparison groups I (for non-traumatic OHCA) and II (for traumatic OHCA) included 1,122 and 63 patients, respectively. Each of them was followed up for 1 year to determine the incidences of newly diagnosed PDDs or other diseases.

### Data Analysis

We used SAS (SAS Institute, Inc., Cary, NC, USA) to select patients from the database. Descriptive statistics, chi-square test, and Cox regression analysis were used in this study. Demographics were classified according to several previous database studies (22, 30). The information included sex (male or female), age (<3, 3–5, 6–9, 10–14, and 15–19 years), family monthly income (>1,000, 600–1,000, and <600, USD$), geographic differences (northern, central, or other location in Taiwan), and the urbanization of the patient's residence (levels 1–4) (31). All descriptive data are reported as the number or percentage. The demographic differences between the survivor and comparison groups were analyzed with the chi-square test. A *p*-value < 0.05 was considered statistically significant. We have analyzed the relative risks for these diseases (between OHCA survivors and comparison patients) using Cox regression analysis. The regression analysis also considered confounding factors of demographics and interactions of diseases. The time from discharge to disease diagnosis was also recorded (<3, 4–6, 7–9, or 10–12 months after discharge).

## Results

### Demographics of the Pediatric OHCA Survivors (*n* = 395)

#### Non-traumatic OHCA Survivors (n = 374)

A total of 2,178 OHCA pediatric patients were classified into the non-traumatic OHCA group. The demographics of the survivor (*n* = 374 survived to discharge) and comparison groups are shown in Table 1. The overall survival rate in the survivor group was 17.2% (374 of 2,178) (Figure 1). The majority of the survivors were male (*n* = 221, 59.1%). Most patients were younger than 3 years (*n* = 173, 46.3%). Most of the population lived in a low-urbanization city (level 4, *n* = 158, 42.2%).

**Table 1:** Demographics of the pediatric OHCA survivors and of the patients in the comparison groups.

**Variables**	**Total 395 OHCA survivors**					
	**Non-traumatic** **(*n* = 374)**	**Comparison group I^**a**^** **(*n* = 1,122)**	***p*-value**	**Traumatic** **(*n* = 21)**	**Comparison group II^**b**^** **(*n* = 63)**	***p*-value**
	**No. (%)**	**No. (%)**		**No. (%)**	**No. (%)**	
**Sex**						
Male	221 (59.1)	663 (59.1)	1.000	13 (61.9)	39 (61.9)	1.000
Female	153 (40.9)	459 (40.9)		8 (38.1)	24 (38.1)	
**Age (years)**						
<3	173 (46.3)	519 (46.3)	1.000	4 (19.0)	12 (19.0)	1.000
3–5	36 (9.6)	108 (9.6)		3 (14.3)	9 (14.3)	
6–9	36 (9.6)	108 (9.6)		0 (0)	0 (0)	
10–14	47 (12.6)	141 (12.6)		4 (19.0)	12 (19.0)	
15–19	82 (21.9)	246 (21.9)		10 (47.6)	30 (47.6)	
**Functional survival**						
Yes	38 (10.2)	–	–	5 (23.8)	–	–
No	336 (89.8)	–		16 (76.2)	–	
**Family income (monthly, USD$)**^**c**^						
>1,100	86 (23.0)	278 (24.8)	0.067	3 (14.3)	16 (25.4)	0.003
600–1,100	202 (54.0)	647 (57.7)		8 (38.1)	39 (61.9)	
<600	86 (23.0)	197 (17.6)		10 (47.6)	8 (12.7)	
**Geographic differences**						
Northern	207 (54.0)	580 (51.7)	0.158	5 (23.8)	34 (54.0)	0.056
Central	86 (23.0)	243 (21.7)		8 (38.1)	14 (22.2)	
Other	81 (21.7)	299 (26.7)		8 (38.1)	15 (23.8)	
**Urbanization**^**c**^						
1 (most)	61 (16.3)	280 (25.0)	0.003	3 (14.3)	20 (31.8)	0.113
2	38 (10.2)	129 (11.5)		0 (0)	5 (7.9)	
3	117 (31.3)	315 (28.1)		8 (38.1)	12 (19.1)	
4	158 (42.2)	398 (35.5)		10 (47.6)	26 (41.3)	

a Comparison group for non-traumatic OHCA patients.

b Comparison group for traumatic OHCA patients.

c *Significant factors*.

#### Traumatic OHCA Survivors (n = 21)

Only 288 children suffered from traumatic OHCA. Their demographics are also shown in Table 1. The survival rate was 7.3% (21 of 288) (Figure 1). Most survivors were male (*n* = 13, 61.9%). The age group comprising patients aged 15–19 years was the most predominant age group (*n* = 10, 47.6%). Most children were in lower-income families (<600 USD$ monthly, *n* = 10, 47.6%).

#### Functional Survivors (n = 43)

The overall functional survival rates were 1.7% (*n* = 38) for the non-traumatic OHCA cases and 1.7% (*n* = 5) for the traumatic OHCA cases.

### Postdischarge Diseases

#### Non-traumatic OHCA Survivors

Among all non-traumatic OHCA survivors, respiratory tract diseases (*n* = 270, 72.2%), gastrointestinal diseases (*n* = 187, 50.0%), neurological diseases (*n* = 167, 49.1%), skin or soft tissue diseases (*n* = 159, 42.5%), and eye/ear diseases (*n* = 99, 26.5%) were the five most common PDD types, followed by psychiatric diseases (*n* = 50, 13.4%), cardiovascular diseases (*n* = 37, 9.9%), and urinary system diseases (*n* = 24, 6.4%). Detailed information regarding the five most common PDD types is shown in Table 2. Pneumonia (*n* = 85, 22.7%), acute gastroenteritis (*n* = 130, 34.8%), and epilepsy (*n* = 77, 20.6%) constituted the majority of respiratory, gastrointestinal, and neurological diseases, respectively. Furthermore, aspiration-related, atypical infections, and influenza were the three most common causes of pneumonia. In most cases, acute gastroenteritis was non-infective (*n* = 88, 23.5%), and epilepsy was focal (*n* = 26, 7.0%). In addition, in comparison group I (*n* = 1,121), allergic rhinitis (*n* = 45, 4.0%), constipation (*n* = 71, 6.3%), tension headaches (*n* = 10, 1.0%), and urticaria/angioedema (*n* = 116, 10.3%) were the most common respiratory tract, gastrointestinal, neurological, and skin or soft tissue diseases, respectively (Table 2).

**Table 2:** The five most common postdischarge disease categories of the non-traumatic OHCA survivors.

**Non-traumatic OHCA survivors** **(*n* = 374)**	**Comparison group I** **(*n* = 1,121)**
Respiratory tract diseases (*n* = 270)	Respiratory tract diseases (*n* = 984)
Pneumonia (*n* = 85)	Acute upper respiratory infection (*n* = 652)
Aspiration-related (*n* = 23)	Acute bronchitis (*n* = 221)
Atypical infection (*n* = 29)	Allergic rhinitis (*n* = 45)
Influenza-caused (*n* = 18)	Asthma (*n* = 36)
Acute upper respiratory infection (*n* = 81)	Others (*n* = 30)
Acute bronchitis (*n* = 71)	
Others (*n* = 33)	
Gastrointestinal diseases (*n* = 187)	Gastrointestinal diseases (*n* = 570)
Acute gastroenteritis (*n* = 130)	Acute gastroenteritis (*n* = 409)
Non-infective (*n* = 88)	Non-infective (*n* = 286)
Infective (*n* = 42)	Infective (*n* = 123)
Ileus (*n* = 18)	Constipation (*n* = 71)
Irritable bowel syndrome (*n* = 17)	Irritable bowel syndrome (*n* = 52)
Peptic ulcer (*n* = 6)	Others (*n* = 38)
Others (*n* = 16)	
Neurological diseases (*n* = 167)	Neurological diseases (*n* = 21)
Epilepsy (*n* = 77)	Tension headache (*n* = 10)
Focal (*n* = 26)	Epilepsy (*n* = 7)
Generalized (*n* = 23)	Others (*n* = 4)
Status (*n* = 15)	
Paralysis (*n* = 32)	
Cerebral palsy (*n* = 26)	
Others (*n* = 32)	
Skin or soft tissue diseases (*n* = 159)	Skin or soft tissue diseases (*n* = 769)
Dermatitis (*n* = 114)	Dermatitis (*n* = 339)
Contact (*n* = 59)	Contact (*n* = 107)
Diaper (*n* = 28)	Atopic (*n* = 82)
Infective (*n* = 18)	Diaper (*n* = 71)
Cellulitis (*n* = 31)	Infective (*n* = 42)
Tinea corporis (*n* = 7)	Others (*n* = 37)
Others (*n* = 7)	Urticaria/angioedema (*n* = 116)
	Furuncles/carbuncle (*n* = 69)
	Others (*n* = 245)
Eye/ear diseases (*n* = 99)	Eye/ear diseases (*n* = 338)
Otitis (*n* = 44)	Conjunctivitis (*n* = 151)
Media (*n* = 34)	Otitis (*n* = 94)
Externa (*n* = 10)	Media (*n* = 61)
Conjunctivitis (*n* = 36)	Externa (*n* = 33)
Hordeolum (*n* = 8)	Hordeolum (*n* = 32)
Visual loss (*n* = 6)	Others (*n* = 61)
Others (*n* = 5)	

#### Traumatic OHCA Survivors

For the traumatic OHCA survivors, the detailed PDDs are listed in Table 3. Respiratory tract diseases (*n* = 17, 81.0%) were the most common, followed by skin or soft tissue diseases (*n* = 14, 66.7%), psychiatric diseases (*n* = 11, 52.4%), neurological diseases (*n* = 9, 42.9%), eye/ear diseases (*n* = 9, 42.9%), gastrointestinal diseases (*n* = 8, 38.1%), and urinary system diseases (*n* = 5, 23.8%). Furthermore, acute URI (*n* = 6, 28.6%), dermatitis (*n* = 6, 28.6%), and attention deficit hyperactivity disorder (*n* = 6, 28.6%) were the more common respiratory tract, skin, or soft tissue and psychiatric diseases, respectively. In comparison group II (*n* = 63), psychiatric (*n* = 3, 4.8%), and neurological (*n* = 3, 4.8%) diseases were not common (Table 3).

**Table 3:** The five most common postdischarge disease categories of the traumatic OHCA survivors.

**Traumatic OHCA survivors** **(*n* = 21)**	**Comparison group II** **(*n* = 63)**
Respiratory tract diseases (*n* = 17)	Respiratory tract diseases (*n* = 52)
Acute upper respiratory infection (*n* = 6)	Acute upper respiratory infection (*n* = 33)
Acute bronchitis (*n* = 5)	Acute bronchitis (*n* = 10)
Pneumonia (*n* = 4)	Allergic rhinitis (*n* = 4)
Others	Others (*n* = 5)
Skin or soft tissue diseases (*n* = 14)	Skin or soft tissue diseases (*n* = 39)
Dermatitis (*n* = 6)	Dermatitis (*n* = 16)
Cellulitis (*n* = 3)	Urticaria/angioedema (*n* = 5)
Tinea corporis (*n* = 3)	Furuncles/Carbuncle (*n* = 4)
Others	Others (*n* = 14)
Psychiatric diseases (*n* = 11)	Psychiatric diseases (*n* = 3)
Attention deficit hyperactivity disorder (*n* = 6)	Anxiety
Borderline personality disorder (*n* = 3)	Developmental disorder
Others	
Eye/ear diseases (*n* = 9)	Eye/ear diseases (*n* = 18)
Peripheral vertigo (*n* = 4)	Conjunctivitis (*n* = 8)
Conjunctivitis (*n* = 3)	Otitis (*n* = 5)
Others	Others (*n* = 5)
Neurological diseases (*n* = 9)	Neurological diseases (*n* = 3)
Paralysis (*n* = 4)	Tension headaches
Hydrocephalus (*n* = 3)	Epilepsy
Others	

*Patient privacy policy of the Taiwanese government: to protect personal privacy, the data are not allowed to be revealed (or marked) if the number of patients is lower than three (potential risk of identification)*.

#### Functional Survivors

Detailed information regarding the functional survivors is presented in Table 4. Neurological (*n* = 14, 36.8%), respiratory tract (*n* = 26, 68.4%), and gastrointestinal (*n* = 15, 39.5%) diseases were the three most common PDD types in non-traumatic OHCA patients. Among the traumatic OHCA cases, respiratory disease (*n* = 5, 100%) was the most common.

**Table 4:** The postdischarge diseases of the functional survivors.

**Non-traumatic OHCA (*n* = 38)**	**Traumatic OHCA (*n* = 5)**
Respiratory tract diseases (*n* = 26)	Respiratory tract diseases (*n* = 5)
Pneumonia (*n* = 10)	Acute upper respiratory infection
Aspiration-related (*n* = 4)	Neurological diseases (*n* = 3)
Atypical infection (*n* = 3)	Hydrocephalus
Acute upper respiratory infection (*n* = 6)	Urinary system diseases (*n* = 3)
Acute bronchitis (*n* = 4)	Urinary tract infection
Neurological diseases (*n* = 14)	Skin or soft tissue diseases (*n* = 3)
Epilepsy (*n* = 7)	Dermatitis
Focal (*n* = 4)	
Generalized/status (*n* = 3)	
Paralysis (*n* = 6)	
Gastrointestinal diseases (*n* = 15)	
Acute gastroenteritis (*n* = 8)	
Non-infective (*n* = 4)	
Infective (*n* = 4)	
Skin or soft tissue diseases (*n* = 10)	
Dermatitis (*n* = 6)	
Contact (*n* = 3)	
Cellulitis (*n* = 3)	
Cardiovascular diseases (*n* = 7)	
Urinary system diseases (*n* = 5)	
Eye/ear diseases (*n* = 5)	
Psychosis diseases (*n* = 4)	

*Patient privacy policy of the Taiwanese government: to protect personal privacy, the data are not allowed to be revealed (or marked) if the number of patients is lower than 3 (potential risk of identification)*.

#### Relative Risk for PDD Between OHCA Survivors and Comparison Patients

We found that only pneumonia (HR 2.52, 95% CI 1.79–3.54) and epilepsy (HR 22.51, 95% CI 10.19–49.69) were significantly higher in OHCA survivors than in comparison patients (data not shown).

### Time Since Discharge to PDD Onset

**Figure 2:**
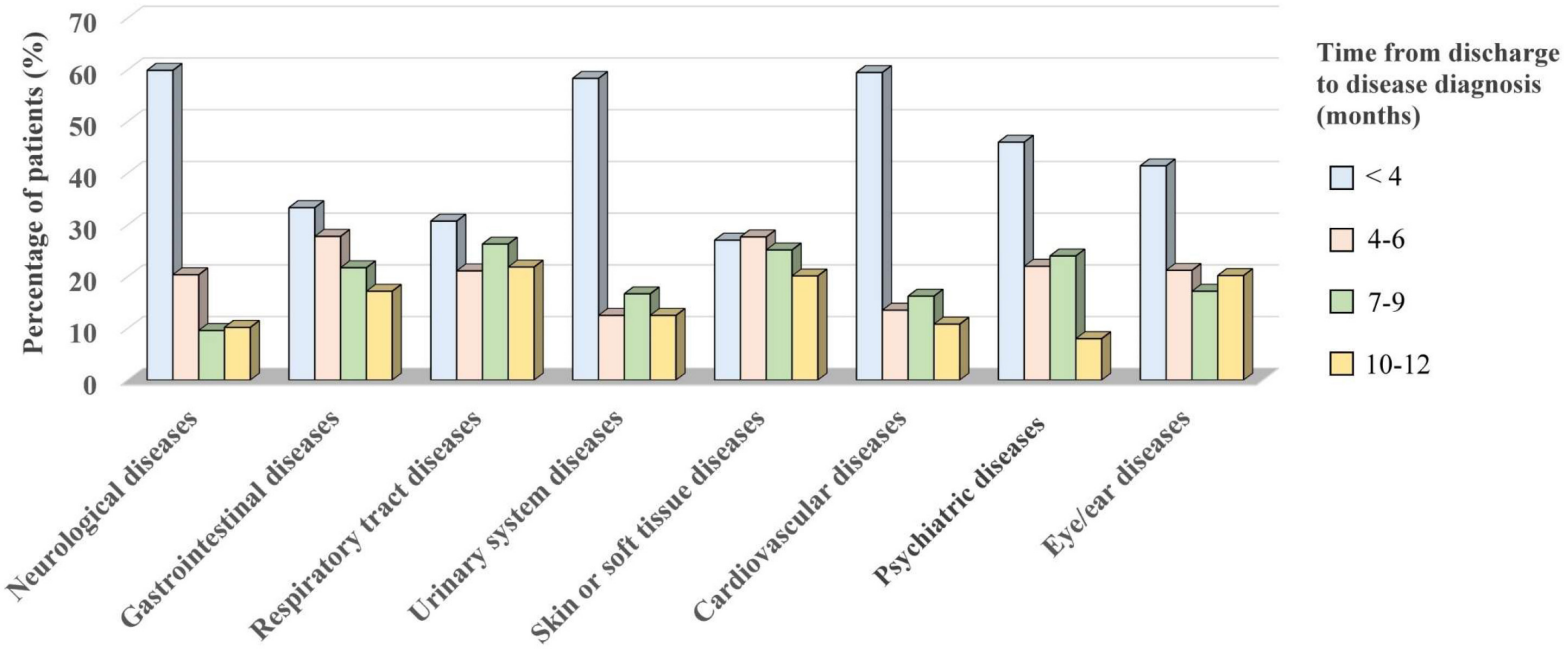
The time from discharge to disease diagnosis in the non-traumatic OHCA survivor group. Most of them were diagnosed within the first 3 months after discharge. The diseases were mainly classified as neurological (*n* = 167), gastrointestinal (*n* = 187), respiratory tract (*n* = 270), urinary system (*n* = 24), skin or soft tissue (*n* = 159), cardiovascular (*n* = 37), psychiatric (*n* = 50), and eye/ear diseases (*n* = 99).

The time between hospital discharge and PDD onset in the non-traumatic OHCA survivor group is shown in Figure 2. Regarding the non-traumatic OHCA survivors, more than half of them had neurological (59.9%), urinary system (58.3%), and cardiovascular diseases (59.5%) within the first 3 months. Skin or soft tissue diseases were more common (27.7%) from 4 to 6 months after discharge. Regarding the traumatic OHCA survivors, most diseases also occurred within the first 3 months after discharge, except for the skin or soft tissue diseases (data not shown).

## Discussion

In this national-population-based study, we have traced pediatric OHCA survivors, reported their demographics, and reported the newly diagnosed PDDs in the groups of pediatric survivors with traumatic and non-traumatic OHCA. The main causes of arrest were obviously different between the two groups, but their demographics were also different. The age distribution was the first key difference. Some population-based studies that have included all pediatric OHCA have concluded that the survival rate is higher for older children or young adults (1, 23, 32–34). Indeed, in this study, we found that almost half (47.6%) of the traumatic OHCA survivors were in the older age group (15–19 years); however, most (46.3%) of the non-traumatic OHCA survivors were younger than 3 years. We recommend that healthcare strategies (i.e., long-term care policy and rehabilitation device designs) for pediatric OHCA survivors be considered not only for older children but also for younger children.

Many studies have tried to identify treatment strategies for increasing the probability of achieving the return of spontaneous circulation, a good survival rate, and functional neurological outcomes (27, 35–37); however, reports of efforts regarding long-term follow-up for pediatric OHCA survivors are few. Some studies, focusing on adult OHCA survivors, have reported that infections, heart failure, and gastrointestinal hemorrhage are the three most common PDDs (22, 38, 39). These diseases in adults are thought to be strongly associated with the causes of cardiac arrest in these adults (21). For example, acute-coronary-syndrome-related cardiac arrest might progress to heart failure, and long-term anticoagulative medications (i.e., aspiration and warfarin) can be responsible for gastrointestinal hemorrhage (21, 22, 40, 41). Since the major etiologies of pediatric OHCA (sudden infant death syndrome and asphyxia) differ from those of adult OHCA (cardiovascular diseases and infections), the PDDs of these two groups are expected to be different (1, 12, 35, 42).

For the non-traumatic OHCA survivors, we found that respiratory tract diseases were the most common problems. Some investigations have mentioned that healthcare-associated pneumonia (i.e., ventilator-associated pneumonia and nosocomial infections) is one of the most common comorbidities in OHCA patients during their hospital stay (13, 43). Moreover, some studies have even recommended that antibiotics be administered as a preventive treatment within the first day after OHCA (43, 44). Unfortunately, treatment recommendations for postdischarge pneumonia are still not clear for survivors. In this study, we first noted that atypical infections (including mycoplasma and legionella) and influenza (total 55.2%) were the major causes of pneumonia after discharge. Moreover, 7.9% (*n* = 3) of functional survivors still suffered from atypical pneumonia. We suggest the use of antibiotics early during the care of patients or at discharge to cover atypical pathogens and that the influenza test not be ignored for patients suffering from postdischarge pneumonia.

Hypoxia/ischemic brain injury is almost unavoidable during the no-flow period of OHCA in patients. Neurological defects are reasonably expected for most survivors. A few studies have followed pediatric survivors and tried to determine risk factors for dysfunctional outcomes (including age and the presence of preexisting conditions) (35, 45). However, except for reports on evaluations of function/dysfunction, detailed reports with further neurological descriptions of pediatric survivors are few. There were only 38 non-traumatic OHCA survivors and 5 traumatic OHCA functional survivors in this study. We noted that epilepsy (20.6%) was one of the most common neurological diseases in all non-traumatic OHCA survivors. In addition, 20.6% (*n* = 77) of the survivors suffered from generalized/status epilepsy and focal epilepsy. More importantly, 18.4% (*n* = 7) of the functional survivors still suffered from epilepsy. Although antiepileptic drugs are not routinely recommended for all pediatric OHCA cases, we recommend that antiepileptic drugs be carefully considered for epilepsy prevention, especially for those with severe hypoxic brain injuries.

Finally, for both traumatic and non-traumatic OHCA survivors, we also noted that most of these diseases (including almost half of the neurological, urinary system, and cardiovascular events) were newly diagnosed within the first 3 months after discharge. Dermatitis was the main skin problem for all survivors (30.5% of non-traumatic OHCA survivors and 28.6% of traumatic survivors), and it was more common at 4–6 months after discharge. Therefore, aggressive follow-up and education for primary caregivers should be emphasized as early as possible.

In conclusion, respiratory tract (pneumonia), neurological (epilepsy), and skin or soft tissue (dermatitis) diseases are very common PDDs in both non-traumatic and traumatic OHCA survivors. More importantly, most PDDs, except for skin or soft tissue diseases, are newly diagnosed within the first 3 months after discharge.

### Limitations

The main limitation of this study was how we defined the PDD onset time. The true onset times of the diseases were difficult to determine clearly in this retrospective database study because the diseases might have already developed before discharge (may not have presented obviously or may not have been recorded). Therefore, we defined these diseases as “newly diagnosed” instead of using the term “new-onset.” The second limitation was the potential errors in the disease classification. For example, if a patient was initially diagnosed with acute URI in the clinic, his/her disease may have progressed to pneumonia with no visit to any hospital for follow-up. This patient was recorded as only having URI. The data regarding the pre-hospitalization information were not included in this database. Finally, the Utstein guidelines recommend that the neurological outcome at hospital discharge be determined by means of specific scales (29). However, the PCPCS was not used as the basis for evaluation of the functional survivors in this study. The major reason was that the evaluation with the PCPCS was not routinely or regularly performed for each survivor after discharge. Finally, the number of traumatic OHCA functional survivors was very small, and the findings for this group might not provide useful data for clinical recommendations.

## Data Availability Statement

All datasets generated for this study are included in the article/supplementary material. Government's permission is required to obtain the datasets. Permission can be obtained from the Health and Welfare Data Center: https://dep.mohw.gov.tw/DOS/np-2497-113.html.

## Author Contributions

T-HL, I-CJ, H-YH, and Y-RL conceived the study. Y-RL, W-LC, C-CH, and M-CY managed the data, including quality control. Y-RL, W-YL, M-CY, C-ML, and T-HL provided statistical advice on the study design and analyzed the data. Y-RL, C-CC, and C-FC chaired the data oversight committee. Y-RL and T-HL drafted the manuscript. Y-RL assumed responsibility for the paper as a whole. All authors contributed substantially to the manuscript revision, read, and approved the final manuscript.

### Conflict of Interest

The authors declare that the research was conducted in the absence of any commercial or financial relationships that could be construed as a potential conflict of interest.
